# Raptor talon shape and biomechanical performance are controlled by relative prey size but not by allometry

**DOI:** 10.1038/s41598-019-43654-0

**Published:** 2019-05-08

**Authors:** Leah R. Tsang, Laura A. B. Wilson, Justin Ledogar, Stephen Wroe, Marie Attard, Gabriele Sansalone

**Affiliations:** 1 Ornithology Collection, Australian Museum Research Institute, Australian Museum, 1 William Street, Sydney, New South Wales 2010 Australia; 2 Function, Evolution and Anatomy Research Laboratory, Zoology, School of Environmental and Rural Sciences, University of New England, Armidale, NSW 2351 Australia; 3 Palaeontology, Geobiology & Earth Archives Research Centre, School of Biological, Earth and Environmental Sciences. University of New South Wales, Sydney, NSW 2052 Australia; 4 Department of Evolutionary Anthropology, Duke University, Durham, NC 27708 USA; 5 Department of Animal and Plant Sciences, The University of Sheffield, Sheffield, S10 2TN UK

**Keywords:** Evolution, Biomechanics

## Abstract

Most birds of prey (raptors), rely heavily on their talons for capturing prey. However, the relationship between talon shape and the ability to take prey is poorly understood. In this study we investigate whether raptor talons have evolved primarily in response to adaptive pressures exerted by different dietary demands, or if talon morphology is largely constrained by allometric or phylogenetic factors. We focus on the hallux talon and include 21 species in total varying greatly in body mass and feeding ecology, ranging from active predation on relatively large prey to obligate scavenging. To quantify the variation in talon shape and biomechanical performance within a phylogenetic framework, we combined three dimensional (3D) geometric morphometrics, finite element modelling and phylogenetic comparative methods. Our results indicate that relative prey size plays a key role in shaping the raptorial talon. Species that hunt larger prey are characterised by both distinct talon shape and mechanical performance when compared to species that predate smaller prey, even when accounting for phylogeny. In contrast to previous results of skull-based analysis, allometry had no significant effect. In conclusion, we found that raptor talon evolution has been strongly influenced by relative prey size, but not allometry and, that talon shape and mechanical performance are good indicators of feeding ecology.

## Introduction

Identifying the factors that drive morphological evolution is a central topic in evolutionary biology^1,2^. Birds use their talons (claws) to perform a wide variety of tasks and their talon morphology has often been associated with a particular behaviour and/or ecology^3–5^. For example, birds claws can show clear adaptations to perching (Passeriformes), climbing (Piciformes), manipulating objects (Psittaciformes), foraging on the ground (Galliformes) and killing/restraining prey (birds of prey, i.e., raptors)^3,4^. The talons of raptors are of particular interest because of their importance in predatory success^4^. Typically raptors possess tapered and markedly curved talons compared to those of other birds^3,5^. However, talon morphology has received little attention, with most studies focused mainly on analysing curvature and using this proxy to quantify behaviour and ecology^3–8^. Some authors have adopted different methods and metrics in order to determine possible associations between claw function and claw geometry^4,5,9,10^. For example, Csermely and Rossi^3^ considered different linear measurements to separate raptorial from non-raptorial species, whereas Fowler and colleagues^4^ considered interdigit size variation to make similar distinctions. A few *in vivo* studies have considered some proxy of mechanical performance to test whether a relationship exists between predatory behaviour and grip forces^9,10^.

However, the analysis of single phenotypic features (e.g., talon curvature, grip strength) might not be able to reveal one-to-one relationships between form and function^2^, because different forms may produce similar performance outputs, as suggested by the “many-to-one” model^11^. In other words, divergent forms may produce similar functional capabilities, thus precluding a single, straightforward relationship between form and function, even if phenotypes have evolved adaptively in response to similar selective pressures^2,12^. Furthermore, other factors that could potentially impact talon morphology, such as evolutionary allometry and phylogenetic history, have remained largely untested. Recently, Bright and colleagues^13^ showed that the raptor beak and skull are highly integrated and controlled by allometric factors more than by dietary ones. This suggests that strong developmental constraints may channel raptor beak and skull shape along specific evolutionary trajectories, challenging the common view of the birds’ beak as a highly adaptable structure^13^. Further, the pervasive presence of evolutionary allometry in the avian lineage was confirmed by Felice and Goswami^14^, who also included non-raptorial taxa in their work.

In the present study, we combined 3D geometric morphometrics (GMM), Finite Elements Analysis (FEA) and Phylogenetic Comparative Methods (PCM) to provide a deeper understanding of raptor talon evolution. These methods can be integrated within the framework of evolutionary biology and their combined application has yielded significant advancements in the understanding of evolutionary dynamics among different clades^15–17^. Here we sampled representatives of each raptorial clade (Accipitridae, Falconidae, Strigiformes and Pandionidae), as well as non-raptorial species (Passeriform, Psittaciform) to determine whether, in a phylogenetic context, the evolution of raptor talon shape and biomechanical performance has been driven by feeding ecology, or whether, as with the avian skull, it has been heavily constrained by allometric factors.

## Results

### Dietary categories

The species included in this study have been classified into the following three broad categories according to the ratio between their average body mass (to account for sexual dimorphism) and maximum prey body mass: relatively small to medium sized prey (SM), assigned to species that are able to capture prey that weighs up to half of their body mass; medium to large sized prey (ML), assigned to species that are able to capture prey weighing from half to more than half of their body mass; non-predatory (NP), assigned to species that don’t use their feet for prey capture. The dietary categories have been defined based on existing literature, summarised in Table 1. Maximum prey body mass values are taken for largest prey items that are regularly taken (total number of captures), as opposed to larger prey that might be taken irregularly or recorded in very low numbers (<5 records).

**Table 1:** Sampling effort and dietary category species assignment.

Dietary category	Species included
SM	*Elanus axillaris, E. caeruleus, Falco berigora, F. cenchroides, F. peregrinus, Hieraaetus morphnoides, Milvus migrans, Ninox boobook, Pandion cristatus, Tyto alba*
ML	*Accipiter cirrocephalus, A. novaehollandiae, Aquila audax, Circus approximans, Haliaeetus leucogaster, Haliastur sphenurus*
NP	*Aegypius monachus, Corvus coronoides, Eolophus roseicapilla, Vultur gryphus*

### GMM talon shape analysis

**Figure 1:**
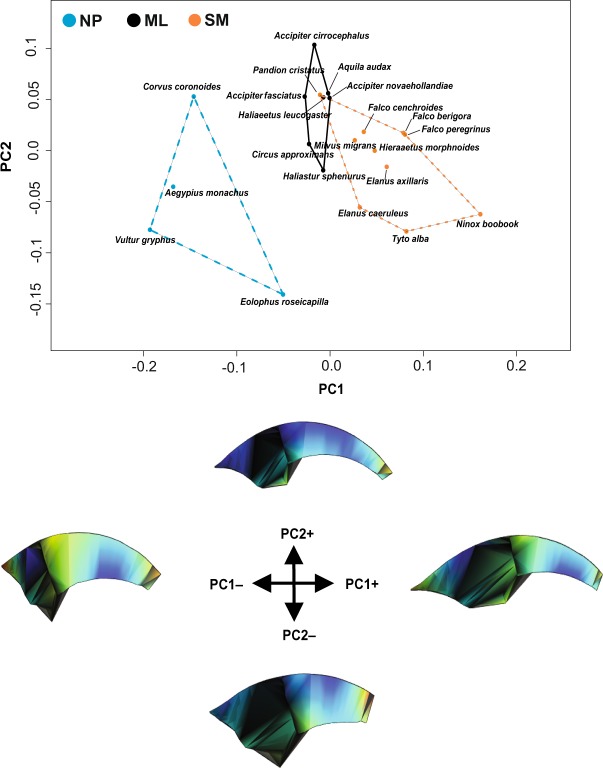
PC1/PC2 scatterplot of the PCA on talon shape variables. Warped meshes refer to positive and negative extremes of the axes. Warped meshes colors refer to the intensity of the shape changes: cooler colours (blue) indicate less change, warmer colours (orange-red) indicate major changes.

Principal Component Analysis (PCA) plots show a clear separation between the three considered dietary categories (see Table 1), i.e., non-predators (NP), and predators on relatively small prey or large prey (SM and ML, respectively) (Fig. 1 and Supplementary Fig. S1). In particular, along the PC1 axis (51.1% of the total variance) the non-predators (NP, vultures, *Corvus* and *Eolophus*) cluster at negative values and are characterized by a less curved, and, overall, shorter talon with a less robust ungual articulation (see Fig. 1). At positive values of PC1 the talon morphology is characterized by a more curved and pointed ungual shaft and by a more robust ungual articulation, representative of species preying on small to medium sized prey (SM). PC2 (24.06% of the total variance) (Fig. 1) separates, from positive to negative values, taxa belonging to medium to large (ML) and small to medium (SM) dietary categories. At positive values of PC2 the talon is characterized by a highly curved morphology and concave articular facet, whereas at negative values the talon is shorter, less curved and characterized by a flatter articular facet. In particular, the ML taxa (positive values), including osprey, the two large eagles (*Aquila, Haliaeetus*), and the Grey and Brown Goshawks, occupy a restricted area of the PC1-PC2 morphospace and are characterized by a longer and more curved talon, whereas the whistling kite (*Haliastur sphenurus*) and the swamp harrier (*Circus approximans*) overlap with the SM taxa and are characterized by a less curved talon, a flatter articular facet and a smaller flexor tubercle. It must be noted that within the SM group there are clear separations between family groups, with Falconidae sharing positive values for both PC1 and PC2, in contrast to the crepuscular Accipitrids (*Elanus*) and the nocturnal owls (*Tyto* and *Ninox*), sharing negative values along PC2.

The Procrustes ANOVA performed on the shape variables confirmed the groups clustering observed in the PCA and returned highly significant results (F_2,21_ = 8.725; r^2^ = 0.478; *p*-value = 0.001). Moreover, pairwise comparisons evidenced that all the dietary categories were significantly different in shape from each other (Table 2).

**Table 2:** Pairwise comparison of shape differences between dietary groups.

Dietary catergory	ML	NP	SM
ML	0	**0.002**	**0.006**
NP		0	**0.002**
SM			0

*P*-values are shown above the diagonal, and significant results (*p* < 0.05) are highlighted in bold. ML = medium to large-sized prey, NP = non-predatory, SM = small to medium-size prey.

### Finite element analysis

**Figure 2:**
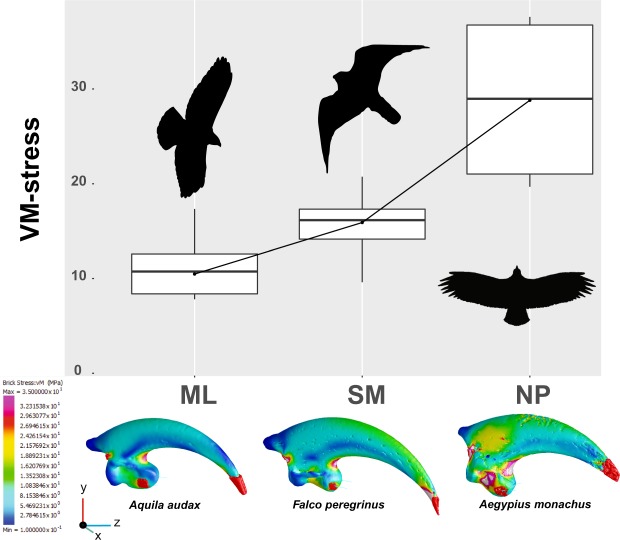
Boxplot of mean VM stress values detected at the 26 homologous landmarks and representative FEMs for the three dietary categories. Bottom and top of the boxes are the first and third quartiles, the horizontal black lines represent the mean and whiskers represent the minimum and maximum values. Animal silhouettes were available under Public Domain license at phylopic (http://phylopic.org/). Specifically, starting from the left, Accipitrinae (http://phylopic.org/image/e00734a7-e8a8-4fe5-b5a9-58d927ca451a/), This image is available for reuse under the Public Domain Dedication 1.0 license; Falconidae (http://phylopic.org/image/6cebf754-cb71-448d-a5bb-947157205264/), available for reuse and under the Creative Commons Attribution 3.0 Unported (https://creativecommons.org/licenses/by/3.0/) image by Liftarn; Cathartiformes (http://phylopic.org/image/901bfc1f-5f97-499c-961f-6cf4d8ab6239/), available for reuse and under the Creative Commons Attribution 3.0 Unported (https://creativecommons.org/licenses/by/3.0/) image by Mark P. Witton & Darren Naish.

When we applied FEA simulation to the talon models (see Materials and Methods for detail on protocols and boundary conditions), we found different stress magnitudes and distributions between the three dietary categories (NP, SM and ML; Fig. 2 shows three representative models and Supplementary Fig. S2 summarizes the FEA results). Non-predatory species (NP), e.g., *Aegypius monachus* display, on average, higher von Mises (VM) stress values that are concentrated on the flexor tubercle, the ventral region of the inner curvature, along the outer curvature, and on the medial edge of the talon. In contrast, species that feed on larger prey items (ML, e.g. *Haliaeetus leucogaster*, *Aquila audax*) showed, on average, the lowest VM stress values in correspondence with those regions previously described (Fig. 2 and Supplementary Fig. S2). In particular, results showed reduced VM stress values on the outer and inner curvature, the medial edge and the flexor tubercle, whereas the ventral region of the inner curvature showed high stress values. Species belonging to the SM category (e.g., *Falco peregrinus*) displayed, on average, intermediate VM stress values. The ventral region of the inner curvature, the flexor tubercle and the outer curvature were markedly more stressed when compared to the taxa belonging to the ML category (Fig. 2 and Supplementary Fig. 2). The ANOVA test performed on the mean VM stress values confirmed the inter-group differences (Fig. 2) returning highly significant results (F_2,21_ = 7.322; r^2^ = 0.568; *p*-value = 0.001). Pairwise comparisons confirmed that each of the dietary categories were significantly different in mean VM stress values from each other (Table 3).

**Table 3:** Pairwise comparison of VM stress differences between dietary groups.

Dietary group	ML	NP	SM
ML	0	**0.003**	**0.014**
NP		0	**0.021**
SM			0

*P*-values are shown above the diagonal, and significant results (*p* < 0.05) are highlighted in bold. ML = medium to large-sized prey, NP = non-predatory, SM = small to medium-size prey.

### Evolutionary allometry

Our tests revealed that shape and size (CS) variables were not significantly correlated for each multivariate regression model considered (Table 4). Similarly, all regressions between the averaged VM stress values and size (CS) were non-significant (Table 4).

**Table 4:** The different multivariate regression models of shape and Von Mises stress on size (CS) tested in the present work.

Regression model	F	r^2^	*p*-value
Shape~size	0.898	0.042	0.416
Shape~size no NP	1.136	0.066	0.305
VM stress~size	1.842	0.084	0.088
VM stress~size no NP	0.959	0.056	0.403

### Phylogenetic comparative methods

**Figure 3:**
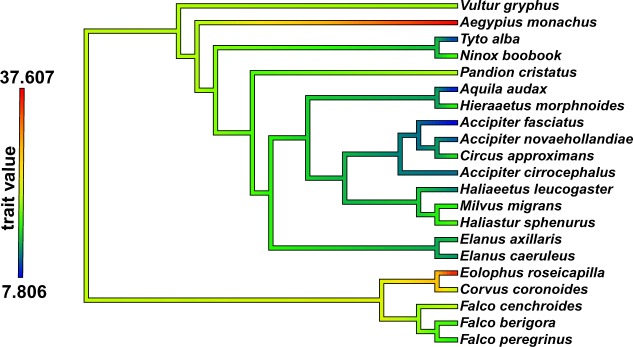
Measured averaged VM stress values for each species mapped on the phylogeny. Values at nodes and branches were reconstructed using a maximum-likelihood ancestral character estimation method based on a Brownian motion model of evolution.

Phylogenetic signal analyses revealed a significant degree of phylogenetic structure in the shape variables (K_mult_ = 0.804, *p*-value = 0.001), and for the averaged VM stress variables (K_mult_ = 1.227, *p*-value = 0.004; see Fig. 3). PGLS analyses revealed that talon shape was not significantly correlated with size (CS) even when excluding the NP taxa (F_1,21_ = 1.321, r^2^ = 0.06; p-value = 0.422; F_1,17_ = 1.147, r^2^ = 0.046; p-value = 0.632), while shape was significantly different between dietary categories (F_2,21_ = 7.148, r^2^ = 0.429, p-value = 0.038). Talon averaged VM stress values were not correlated with size even when excluding the NP taxa (F_1,21_ = 1.741; r^2^ = 0.083; *p*-value = 0.298; F_1,17_ = 1.628; r^2^ = 0.071; *p*-value = 0.343, respectively), whereas they were significantly different between dietary categories (F_2,21_ = 7.174; r^2^ = 0.44; p-value = 0.027).

## Discussion

Our results indicate how talon shape and biomechanical performance evolved in response to the selective pressures exerted by different feeding ecologies, even when phylogeny is taken into account. The maximum prey size relative to body mass proved to be an important factor in shaping the raptor talon. Non-predatory species (NP category) possess shorter, blunter and less curved talons, with flatter articular facets and reduced flexor tubercles, consistent with their unspecialized feeding behaviour. This suggests that the talons of NP species are not constrained by the same biomechanical and morphological influences as predatory species. In fact, NP species are characterized by markedly higher VM stress values (Figs 1 and 2). Vultures are specialized carrion feeders, known to have shorter talons unsuitable for prey seizing^18,19^.

The recurved talon of the galah (*Eolophus roseicapilla*), despite its superficial resemblance to those of raptors^20^, displayed extremely high VM stress values, evidencing that it is poorly adapted to sustain the relatively heavy loads applied during prey capture.

Conversely, predatory species hunting larger prey (accipitrids, ML category) show highly curved talons, suggesting an adaptation to immobilise and subdue relatively larger and heavier prey^4,18^. Species belonging to the ML category also possessed an enlarged flexor tubercule, suggesting the presence of relatively larger and more powerful digit flexor muscles responsible for transmitting and maintaining grip forces^9,21^. Consistent with these observations, we found that taxa preying on relatively larger species (ML) showed markedly lower VM stress values along the talon curvature and in correspondence with the flexor tubercle, thus suggesting adaptation to sustain a stronger and extended (in time) restraining action^4,9,10^.

Our results showed that the talons of species preying on relatively smaller prey (SM category) were morphologically distinct from those of predators on relatively large prey (ML), being characterized by a slender, less curved talon with a reduced flexor tubercle (Fig. 1). Along PC2 we observed an overlap between the SM species and the whistling kite and the swamp harrier (*H. sphenurus* and *C. approximans*, see Fig. 1 and Supplementary Fig. S1). Despite being able to hunt relatively large prey, these species display more generalised behaviours than the other ML species. *H. sphenurus* also relies on carrion during the breeding season. This is in line with the biomechanical analysis, which showed that the whistling kite and the swamp harrier display higher stresses along the talon curvature and on the flexor tubercle.

However, the osprey (*Pandion cristatus*) overlapped with species belonging to the ML category along the first two PC scores (Fig. 1), although it was clearly separated on the third (Supplementary Fig. S1). This result is in line with other studies which have showed the osprey to have a distinctive morphology, thought to be a consequence of its highly specialized piscivorous diet^4,5,13^. Indeed, the osprey showed a slender, highly curved talon with a much reduced, elongated flexor tubercle characterized by significantly higher VM stress values, comparable to those of the other species belonging to the SM category (Figs 1 and 2).

Despite having highly distinctive talon morphology, the barn owl (*Tyto alba*) showed low VM stress values (Supplementary Table S1 and Fig. S2) comparable with the members of the ML category. This finding may reflect that predicted by the ‘many-to-one’ hypothesis, i.e., that very different forms may have similar functional outputs^11,22^. However, it must be noted that this species possesses a large flexor tubercle (Supplementary Fig. S2) and that, in general, Strigiformes show a highly specialized tendon locking mechanism, short tarsometatarsus and sesamoid bones which contribute to enhance the grip force^4,21,23^. By contrast, the boobook (*Ninox boobook*) displayed average VM stress values in line with the other taxa belonging to the SM category (Supplementary Table S1). The boobook has a more insectivorous diet, possesses a reduced flexor tubercle and a flatter talon, suggesting a lesser degree of specialization toward improved grip force^24^. Furthermore, true owls (Strigidae) are reported to be more specialized toward aerial prey capture, having a tendon locking mechanism more similar to that of falcons than to the closely related barn owls (Tytonidae)^24^.

Among the species included in the SM category, falconids occupied a distinct region of the talon morphospace (Fig. 1). This is in line with previous studies, which have found that falcons rely more on their beak to kill their prey and, therefore, display higher bite forces but weaker grip forces compared to accipitrids^4,5,10,18,25^. In fact, falcons showed higher stress values along the talon curvature, on the medial edge and on the flexor tubercle (Fig. 2). The shape and biomechanical analyses depict a scenario of morpho-functional convergence driven by relative prey size, more than prey type. In fact, species belonging to the same dietary category occupied a similar morphospace and experienced similar VM stress distribution patterns on the talon (Fig. 2). This result is also supported by the absence of correlation between both talon shape and biomechanical performance with size.

Despite a great range of body sizes, the talons of raptors showed similar adaptive features which grouped according to relative prey size, not the absolute size of the predator itself. In other words, the raptor talon is more likely shaped by adaptive factors than developmental constraints, such as allometry. Accipitrids, falconids, Strigiformes and cathartids were separated on the morphospace plot and showed different VM stress values (Figs 1 and 2), which suggests that phylogenetic history may influence talon phenotypes. Further, we found a strong phylogenetic signal in both shape and VM stress variables (Fig. 3). However, the absence of an allometric relationship, when accounting for phylogenetic effect, suggests that the observed variation in both shape and performance cannot be reduced to shared ancestry alone, or the tendency of related species to display similar phenotypes. Therefore, it is likely that raptor talon morphology and performance vary in response to functional selective pressures, since raptor talons are critical for prey capture, killing and/or restraining^4,9,26^.

Our results appear to contrast with those obtained for raptor skulls^13^, where allometry and phenotypic integration were found to have a major impact in shaping the cranial variability of raptors. Although, it must be noted that allometric and heterochronic changes are important factors in the evolution of the vertebrate skull^27–29^. These processes are thought to act as paths of least resistance in the skull’s evolutionary history and may facilitate morphological evolution along particular trajectories^27,30^. However, traits constrained along narrow evolutionary trajectories are also less responsive to changes in selective pressures^31–34^. In this context, the allometry-free evolution of the talon could have allowed raptors to exploit different resources and to expand their ecological niches. Poor correlation between claw curvature and body mass has been evidenced for Aves, Squamata and Mesozoic dinosaur^5^, suggesting that the lack of allometric constraint on claws may also be common to these taxa. Therefore, the evolutionary plasticity of the talon could have had an important role in providing the opportunity for raptors to exploit resources and adaptive pathways that could not have been accessed through changing skull shape, because of the high levels of constraint on this structure^1,13,35^. Our study supports the hypothesis that evolutionary mosaicism has played a key role in the successful adaptive radiation of birds^14^, and that the raptorial talon evolved under different adaptive constraints.

## Materials and Methods

### Specimen collection

In this study, we compared the talon shape and biomechanical performance of 12 species of accipitrid raptors, three species from Falconiformes, and two species of Strigiformes. We also include non-predatory species, comprising a representative of passeriform (raven), parrot (cockatoo), and two vultures (Table 1 and Supplementary Table ST1). Although the keratin sheath reflects actual claw curvature^36^, we chose to focus on the ungual cortical bone as this is the underlying supporting structure of the keratin and the site of attachment for tendons of the major flexor muscles (e.g. *M. flexor hallucis longus*). Most FEA of biological structures treat bone as a simplified, non-porous material, however, our Finite Element Models incorporate geometry of the trabecular networks, which can provide added insight into mechanical performance^37,38^. Ungual bones were sourced from both prepared, disarticulated skeletal material from the Australian Museum; and whole, thawed, unprocessed digits (dissected limbs) at the University of New England, Australia.

### CT-scanning, image processing and finite element model assembly

Micro-computed tomography (µ-CT) scans of ungual bones were taken using a Phoenix GE micro-CT scanner. Specimens were scanned using energy settings set at 110 kV voltage and 140 µA current. Each scan comprised 1000 slices. Images were stacked and processed using GE Phoenix proprietary software. Three-dimensional surface and volume meshes were constructed from each CT scanned talon using Mimics (v.16.0) and 3-Matic (v.8.0) (Materialise N. V.), using previously described protocols^37,39^.

All volume models comprised of between 2 million to 2.1 million tetrahedral elements and between 419,033 to 441,598 nodes (i.e., tetrahedral vertices). The volume models were imported into Strand7 (v.2.4.6) and finite element models were created for each specimen. We note that, as with most similar interspecies comparisons, FEA is used here in a wholly comparative fashion. In this study we aim to compare FE models in the context of their phylogenetic relationships and we applied the same approximations for all the models. Material properties were assigned for cortical bone (Young’s Modulus of 21,100 MPa)^7^. We applied the same properties used by Manning *et al*.^7^ in their study on theropod claw biomechanics and all materials were treated as isotropic, using a Poisson’s Ratio value of 0.32 for bone. To remove the size information, as recommended for comparative and biomechanical shape analyses^15,40,41^, all simulations were performed under a scale-free framework by scaling all the models to the same surface area. The models were scaled using the following relationship: $=\frac{\sqrt{SA}}{SB}$; where K is the scaling factor, SA is the surface area of the target model and SB is the surface area of the reference model.

### Restraints

An elastic solid mesh consisting of beam elements (Structural Steel AS 4100–1998, Young’s Modulus of 200,000 MPa)^39^ was placed on the surface of the model around the selected restraint areas to minimize the incidence of artefacts that can occur where single beam nodes are loaded^42^.

**Figure 4:**
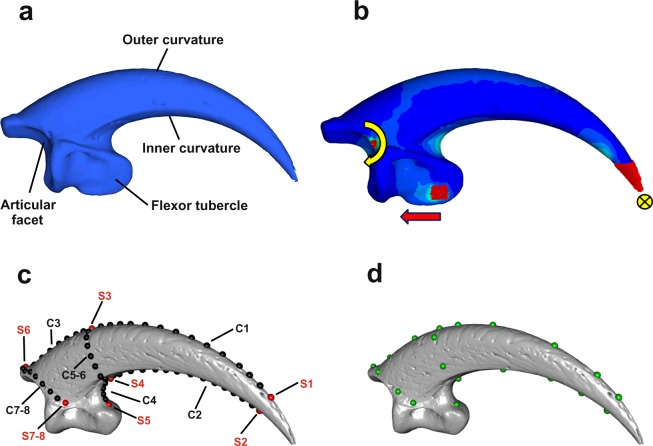
(**a**) 3D model of *Aquila audax* talon showing the anatomical terminology used in the present study. (**b**) Finite element model (FEM) of *Aquila audax* talon, showing location of tessellated beams (red), rotation points (curved yellow arrow), node force location and direction (red arrow) and fixed talon tip (yellow circle). (**c**) The landmarks (red) and semi-landmarks on curves (black) digitized on the ungual bone (*Accipiter cirrhocephalus*). Landmarks and curves definition: *S1-S2*: Tip of the talon. *S3*: talon midpoint height. *S4*: Ventral end of the inner curvature. *S5*: Tip of the flexor tubercle. *S6*: Tip of the extensor tubercle. *S7-8*: Ventral foramina. *C1*: Distal outer curvature. *C2*: Inner curvature. *C3*: proximal outer curvature. *C4*: Ventral curvature. *C5-6*: Talon maximum width. *C7-8*: Lateral borders of the articular facet. (**d**) The selected landmarks (green) used to collect the VM stress values.

All the models were restrained at the tip of the ungual bones for each degree of freedom in translation and rotation. Two restraints were placed respectively at nodes on the apex of the rim of the medial and lateral cotylae (that comprise the articular facet at the proximal end of the ungual bone). These were fixed in translation for each degree of freedom (*x*, *y*, *z*), but left free in rotation along the *x* axis following previous protocols^43^ (see Fig. 4a,b).

The grid boundary of the previously described mesh applied on the distal tip of the ungual bone was identified by visually following the dorsal aspect of the bone, with the grid applied at the distal-most point of the bone tip, then moving around the circumference of the model surface until a conical shaped grid covered the bone tip.

### Digit force estimations and loadings

To assess the biomechanical performance of the ungual bone during flexion, relative forces need to be applied to simulate gripping action for each Finite Element Model (FEM). Forces were applied in the negative direction along the *z* axis.

We used the brown goshawk (*Accipiter fasciatus*) as reference model for scaling the Finite Elements models, using the relationship described in the “CT-scanning, image processing and finite element model assembly” section. This choice is motivated by the similarity in shape and size between the brown goshawk and the Cooper’s hawk (*Accipiter cooperi*) for which the grip force has been measured *in vivo* (9.77 N)^10^. Therefore, the maximum grip force of 9.77 N, estimated for the Cooper’s hawk^10^, was used as the force that was applied to all of the models. As this study focuses on a single talon (hence digit), we calculated the theoretical force equally distributed across all four digits. Although this approach provides a conservative estimate of force applicable to the hallux, the analysis can still be functionally informative from a predatory ecology perspective, since the action of constriction of the feet and toes via muscle flexion produces force output across all toes and talons, which enables a bird to grasp an object. The loads were applied on two nodes placed on the beams composing the elastic mesh embedded respectively on the medial and lateral side of the flexor tubercle. Both nodes were restrained for each degree of freedom (global *x*, *y*, *z* restraint for translation and rotation) and forces applied along the *z* axes. The site of attachment of *M. flexor hallucis longus*, the primary muscle that activates the associated tendon resulting in flexion of the hallux^44^ was, again, covered with an elastic mesh composed by beam elements (Structural Steel AS 4100–1998, Young’s Modulus of 200,000 MPa)^39^, on both the medial and lateral sides of the flexor tubercule, and placed caudo-ventral to the ventral foramina (see Fig. 4a,b), in order to minimize the incidence of artefacts that can occur where single nodes are loaded^42^. To simulate talon flexion (hence piercing action), we applied negative force values at each node on the flexor tubercule, in the negative direction to the ungual tip.

### Von mises stress analysis

To allow a direct comparison of results between talon FEMs, and to further integrate stress and shape outputs from FEA with GMM, we followed the method used in^45^. At the location of 26 selected landmarks (green landmarks on Fig. 4d) sampled from the GMM dataset (see below), we computed the mean von Mises stress values from the four nearest tetrahedral elements (each tetrahedral element has four nodes). After this process was completed, we averaged the landmark VM stress values and the significance of the observed stress variation between SM, ML and NP categories was evaluated by performing an ANOVA on the stress values using the function procD.lm(). Pairwise comparisons between ML, SM and NP categories were performed using the function advanced.procD.lm(). VM stress differences between dietary categories were visualized using a boxplot (Fig. 2).

### Geometric morphometric (GMM) shape analysis

On each talon (Fig. 4a), we digitized 8 anatomical landmarks and 65 semi-landmarks placed equidistantly along the outer and inner curve of the ungual bone to capture its shape in three dimensions (Fig. 4c) using IDAV Landmark software^46^. We manually digitized the homologous landmarks on all the specimens included in this this study, then we manually digitized the semi-landmarks on curves on the *Accipiter fasciatus* talon and used it as the template individual. The points on the template were projected onto all the other specimens following the automatic procedure described in^47^ (the protocol is fully described in Supplementary Methods). Once all the semi-landmarks were automatically placed we imported the landmarks into R version 3.4.4 for further analyses. We performed Generalized Procrustes Analysis^48,49^ (GPA) on all landmarks, implemented in the ProcSym() function from the R package “Morpho”^50^, to rotate, translate, and scale landmark configurations to unit centroid size^51^ (CS, square root of squared differences between landmark coordinates and Centroid coordinates). To visualize the multivariate ordination of the aligned Procrustes coordinates, we performed a principal component analysis (PCA). The significance of the observed shape changes between the three dietary categories was evaluated by performing a Procrustes ANOVA^49^ on aligned Procrustes coordinates using the function procD.lm() from the R package “geomorph”^52^. Pairwise comparisons between ML, SM and NP categories were performed using the function advanced.procD.lm()^52^.

### Evolutionary allometry

We tested the relationship between talon size (independent variable) and Procrustes shape coordinates (dependent variable) by performing a multivariate regression of shape on size (CS). This analysis was performed, and visualized, using the function procD.allometry() from the R package “geomorph”^52^. This analysis was repeated removing the species belonging to the NP category since these taxa could represent potential outliers due to their different ecology and behaviour. These analyses were then repeated using the averaged VM stress values as dependent variables.

### Phylogeny and comparative methods

A maximum clade credibility tree of the species in the analysis was constructed from a set of 1,000 molecular trees^53^ (birdtree.org) using the function MaxCredTree() from the R package “phangorn”^54^.

Phylogenetic signal was calculated for the shape data using the K_mult_ statistic, a method that measures the similarity of trait values in relation to a Brownian motion model of evolution, and that is specifically designed for the challenges of working with high-dimensional landmark configurations^55^. We used a phylogenetic generalized least squares (PGLS) linear model to account for the non-independence among observations due to shared phylogenetic history^56,57^. PGLS linear model and K_mult_ statistic were also computed for the averaged VM-stress values and, then, mapped on the phylogeny using the contMap() function from the R package “phytools”^58^ (Fig. 3).

## Supplementary information

Supplementary information

## Data Availability

The datasets used in the current study are available in the e-publications@UNE repository at http://e-publications.une.edu.au/1959.11/23363. Data can be accessed pending a reasonable request to the corresponding author at the following e-mail address: gsansalone@uniroma3.it.
