# Supplementary material for: Raptor talon shape and biomechanical performance are controlled by relative prey size but not by allometry
Source: Sci Rep. 2019 May 8;9:7076. doi: 10.1038/s41598-019-43654-0 (PMC6506530; doi:10.1038/s41598-019-43654-0)
Supplement: Supplementary file 1 — Supplementary information [file 41598_2019_43654_MOESM1_ESM.pdf]

Supplementary Material to:

Raptor talon shape and biomechanical performance are controlled by relative prey size but not by allometry

Leah R. Tsang, Laura A.B. Wilson, Marie Attard, Justin Ledogar, Stephen Wroe, Gabriele Sansalone

Supplementary materials and methods

Supplementary Table ST1. Species analysed in this study and dietary groupings based on literature review of preferred prey and dietary studies, and relative averaged VM stress values. Diet categories are: SM = small to medium sized prey; ML = medium to large sized prey.

Common and scientific names	Dietary category	Average Body Mass (g)	Maximum Prey Body Mass (g)	References	Average VM stress
Andean condor<br>*Vultur gryphus*	NP	12550	/	Bright et al., (2016)	19.688
Eastern Osprey<br>*Pandion cristatus*	SM	2100	872	Clancy (2005); Marchant & Higgins (1993)	20.253
Black winged Kite<br>*Elanus caeruleus*	SM	290	89	Mendelsohn & Jaksic (1989); Manaa et al. (2013)	12.365
Black-shouldered Kite<br>*Elanus axillaris*	SM	300	97	Debus et al. (2006); Tsang et al. (2017)	13.607
Cinereous Vulture<br>*Aegypius monachus*	NP	9625	/	Bright et al., (2016)	37.601
Little Eagle<br>*Hieraaetus*	SM	815	349	Olsen et al. (2010); Olsen et al. (2013b)	16.247

morphnoides					
Wedge-tailed Eagle<br><br>*Aquila audax*	ML	5725	13475	Olsen et al. (2006); Olsen et al. (2013b)	8.572
Grey Goshawk<br><br>*Accipiter novaehollandiae*	ML	650	1500	Olsen et al. (1990).	10.449
Brown Goshawk<br><br>*Accipiter fasciatus*	ML	525	1500	Aumann (1988); Beranek (2017)	8.805
Collared Sparrowhawk<br><br>*Accipiter cirrhocephalus*	ML	228	205	Czechura et al. (1987)	11.023
Swamp Harrier<br><br>*Circus approximans*	ML	1100	1500	Baker-Gabb (1984)	15.363
Black Kite<br><br>*Milvus migrans*	SM	725	225	Marchant & Higgins (1993);Zawadzka (1999)	16.063
Whistling Kite<br><br>*Haliastur sphenurus*	ML	907	606	Olsen et al. (2013b)	15.337
White-bellied Sea-eagle<br><br>*Haliaeetus leucogaster*	ML	3815	2467	Olsen et al. (2006) Debus (2008); Olsen et al. (2013a)	11.662
Eastern Barn Owl<br><br>*Tyto alba*	SM	565	134	Debus et al. (2004)	9.709
Southern Boobook<br><br>*Ninox boobook*	SM	335	18	Trost et al. (2008)	17.454
Nankeen Kestrel<br><br>*Falco cenchroides*	SM	195	75	Starr et al. (2004); Tsang et	20.745

				al. 2017;	
Brown Falcon<br><br>*Falco berigora*	SM	760	170	McDonald et al. (2012)	17.935
Peregrine Falcon<br><br>*Falco peregrinus*	SM	1000	335	Olsen et al. (2008)	16.783
Galah<br><br>*Eolophus roseicapilla*	NP	353	/	Taylor and Parkin (2008)	36.459
Australian Raven<br><br>*Corvus coronoides*	NP	650	/	Iwaniuk and Arnold (2004)	22.473

Automatic landmarking procedure

The points on the template (*Accipiter fasciatus*) were projected on all the other specimens using the function placePatch() from the R package 'Morpho' (Schlager, 2014). In order to remove any incorrect projection the semi-landmarks on the curves were set bold-distanced using the function pointsOnBezier() from the 'bezier' R package (Olsen, 2015), further the curves present on the sides of the talon geometry were mirrored using the function symmetrize from the R package 'Morpho'. After this process was complete the semi-landmarks were slid along the curves by minimising the bending energy of a thin plate spine deformation (semi-landmarks relaxation) using the slider3d() function from the R package 'Morpho' (Schlager, 2014). This approach follows the algorithm described in Gunz et al. (2005) and has been shown to be the most appropriate method to slide semi-landmarks on curves and surfaces according to Bookstein (2015). All the semi-landmarks are enclosed by homologous landmarks (Gunz, 2005).

Body size proxy

GMM also returns the Centroid Size, an index that permits to get back the information related to size that are removed by GPA. We regressed the centroid size (CS) and body mass estimates taken from the literature (Table ST1), to assess whether CS can be used as proxy for body size. The regression is highly significant (r^2= 0.861, *p*-value=0.001).

Supplementary results

Shape analysis

Along the PC3 (7.35% of the total variance) it is evident the separation, at negative values, of the fishing eagle (*Pandion cristatus*) from the other taxa. The osprey is characterized by a slender and extremely curved talon when compared to the other eagles with which shares similar values of the PC1. However, at positive values of the PC3 it is possible to separate *Circus approximans* from the other ML taxa. *Circus* shares

similar PC3 values with the Cinereous vulture (*Ae. monachus*), indicating that these two species have shorter and robust talon shaft, with the vulture showing a much lesser degree of curvature.

Supplemetary figure S1. PC1/PC3 scatterplot of the PCA on talon shape variables. Warped meshes refer to positive and negative extremes of the axes. Warped meshes colors refer to the intensity of the shape changes: blue indicates less change, red indicates major changes.

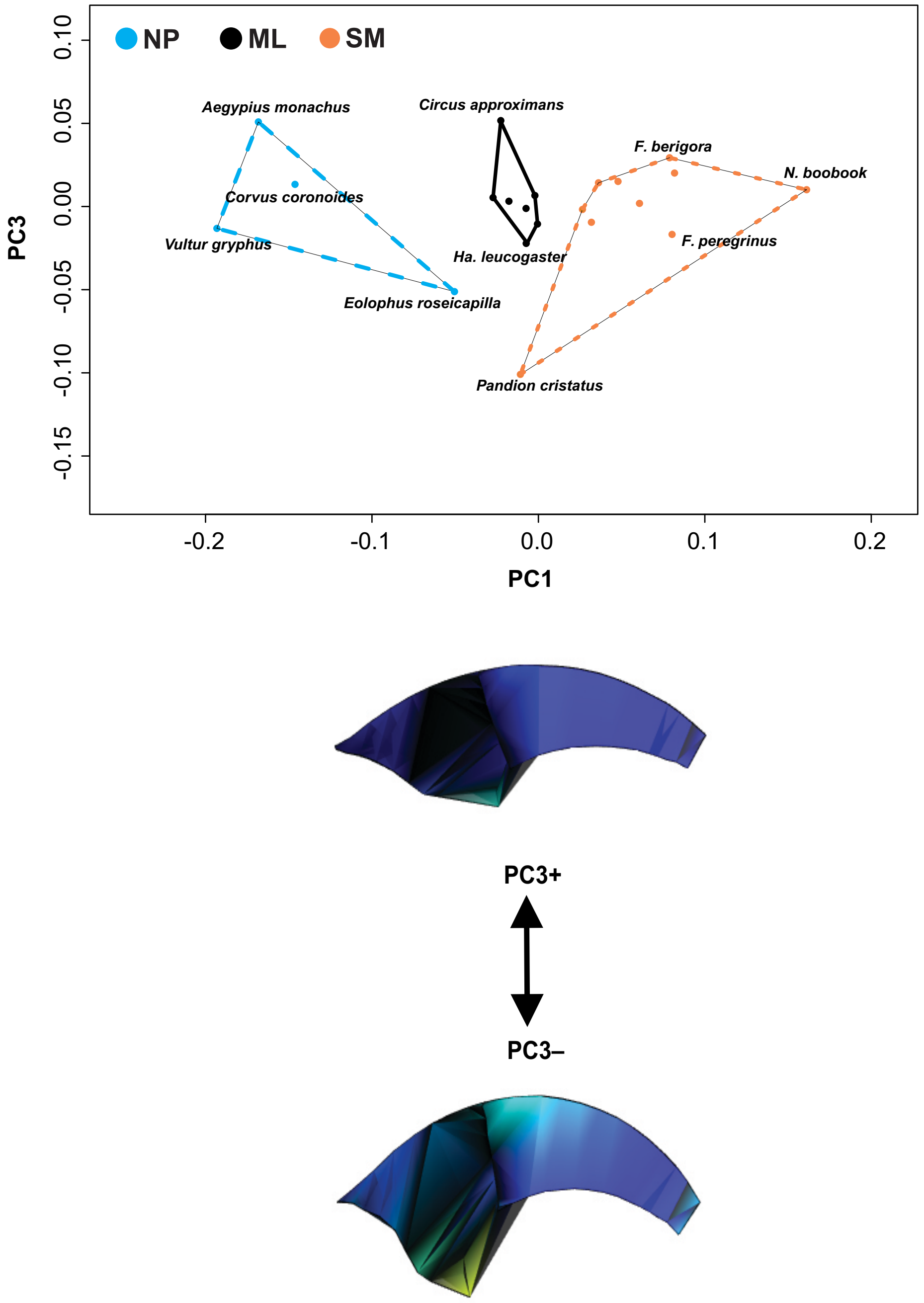

Supplementary FEM results

Supplementary Figure S2. Finite element models in color showing the intensity of stress experienced by the different structures. The three groups are depicted under the same stress scale of von Mises stress. Red colour indicates larger stress.

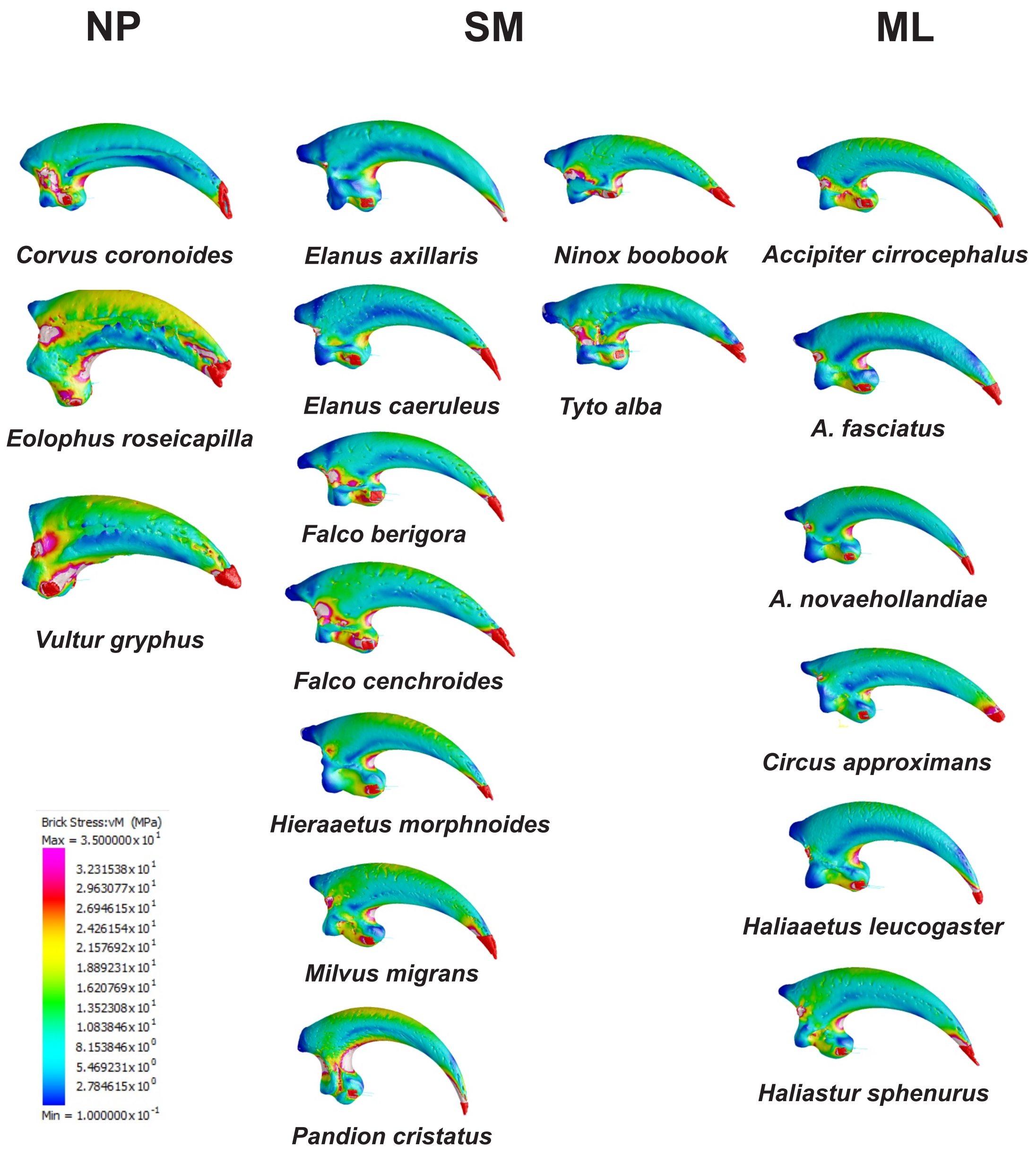

Supplementary References

Aumann, T. (1988) The Diet of the Brown Goshawk, Accipiter fasciatus, in South-eastern Australia. Australian Wildlife Research, 15(6): 587 - 594.

Baker-Gabb, D. (1984) The feeding ecology and behaviour of seven species of raptor overwintering in coastal Victoria. Australian Wildlife Research, (11): 517-532.

Bright, J.A., Marugan-Lobon, J., Cobb, S.N., and Rayfield, E.J. (2016) The shapes of bird beaks are highly controlled by nondietary factors. PNAS, 113 (19) 5352-5357. https://doi.org/10.1073/pnas.1602683113.

Clancy, G. (2005) The diet of the Osprey (Pandion haliaetus) on the north coast of New South Wales. Emu (105), 87-91.

Czechura, G., Debus, S., and Mooney, J. (1987) The Collared Sparrowhawk *Accipiter cirrocephalus*: A Review and Comparison with the Brown Goshawk *Accipiter fasciatus*. Australian Field Ornithology (12)2, 35-62.

Debus, S.J.S. and Rose, A.B. (2004b). Diet of the Barn Owl *Tyto alba* near Tamworth, New South Wales. Corella 28(4): 95.

Debus, S.J.S., Olde, G.S., Marshall, N., Meyer, J., and Rose, A.B. (2006) Foraging, breeding behaviour and diet of a family of Black-shouldered Kites Elanus axillaris near Tamworth, New South Wales. Australian Field Ornithology, (23): 130-143.

Debus, S.J.S. (2008b) Biology and diet of the White-bellied Sea-eagle Haliaeetus leucogaster breeding in northern inland New South Wales. Australian Field Ornithology, (25)4, 165-193.

Iwaniuk, A. & Arnold, K. (2004). Is Cooperative Breeding Associated With Bigger Brains? A Comparative Test in the Corvida (Passeriformes). Ethology. 110. 10.1111/j.1439-0310.2003.00957.x.

McDonald, P.G., Olsen, J., and Rose, A.B. (2012). The Diet of Breeding Brown Falcons (Falco berigora) In the Canberra Region, Australia, with comparisons to other regions, Journal of Raptor Research, 46(4):394-400.

Manaa, A., Souttou, K., Sekour, M., Bendjoudi, D., Guezoul, O., Baziz-Neffah, F., Doumandji, S., Stoetzel, E., and Denys, C. (2013) Diet of Black-shouldered Kite Elanus caeruleus in a farmland area near Algiers, Algeria, Ostrich, 84:2, 113-117, DOI: 10.2989/00306525.2013.781551

Marchant, S., and Higgins, P. J. (1993) Handbook of Australian, New Zealand and Antactic Birds. Volume 2: Raptors to Lapwings. Oxford University Press, Melbourne.

Mendelsohn, J. M. & Jaksić, F. M. (1989) Hunting behaviour of Black-shouldered Kites in the Americas, Europe, Africa and Australia. Ostrich 60(1), 1-12.

Olsen, J. E., Fuentes, E.J., and Rose, A.B. (2006). Trophic relationships between neighbouring White-bellied Sea-Eagles Haliaeetus leucogaster and Wedge-tailed Eagles Aquila audax breeding on rivers and dams near Canberra. Emu (106), 193–201.

Olsen, J. E., Fuentes, E.J., Bird, D.M., Rose, A.B., and Judge, D. (2008) Dietary shifts based on prey availability in Peregrine Falcons and Australian Hobbies near Canberra, Australia. Journal of Raptor Research (42), 125–137.

Olsen, J., Judge, D., Rose, A.B., and Debus, S.J.S. (2010) Diets of Wedge-tailed Eagles Aquila audax and Little Eagles Hieraaetus morphnoides breeding near Canberra. Journal of Raptor Research, 44(1), 50-61

Olsen, J., Debus S.J.S., Rose, A.B., Judge D. (2013a) Diets of White-bellied Sea-Eagles Haliaeetus leucogaster and Whistling Kites Haliastur sphenurus breeding near Canberra, 2003-2008. Corella (37), 13-18.

Olsen, J., Debus, S.J.S., Judge, D., and Rose, A.B. (2013c) Diets of Wedge-tailed Eagles Aquila audax and Little Eagles Hieraaetus morphnoides breeding near Canberra, 2008-2009. Corella, (37), 25 - 29.

Olsen, P.D., Debus, S.J.S., Czechura, G.V., and Mooney, N.J. (1990) Comparative feeding ecology of the Grey Goshawk *Accipiter novaehollandiae* and Brown Goshawk *Accipiter fasciatus*. Australian Bird Watcher 13:178-192.

Starr, M.J., Starr M., and Wilson, S.C. (2004) Hunting rates and prey of a pair of breeding Nankeen Kestrels Falco cenchroides near Sydney, New South Wales. Australian Field Ornithology (21) 71-75.

Taylor, T. D. and Parkin, D. T. (2008), Sex ratios observed in 80 species of parrots. Journal of Zoology, 276: 89-94. doi:10.1111/j.1469-7998.2008.00476.x

Trost, S., Olsen, J.E., Rose, A.B., and Debus, S.J.S. (2008) Winter diet of Southern Boobooks Ninox novaeseelandiae in Canberra 1993–2004. Corella (32), 66–70.Tsang, L.R., Rose, A.B., Fuentes, E.J., Olslen, J., Trost, S., and McDonald, P.G. (2017) A comparison of the diets of the Black-shouldered Kite *Elanus axillaris* and Nankeen Kestrel *Falco cenchroides* in the Canberra region. Corella (41), 27-31.

Zawadzka, D. (1999) Feeding habits of the Black Kite Milvus migrans, Red Kite Milvus milvus, White-tailed Eagle Haliaeetus albicilla and Lesser Spotted Eagle Aquila pomańna in Wigry National Park (NE Poland). Acta Ornithologica (34), 65-75.
